# Identifying Racial Minorities' Nationality: Non-verbal Accent as a Cue to Cultural Group Membership

**DOI:** 10.3389/fpsyg.2021.608581

**Published:** 2021-06-17

**Authors:** Yvette D. Alcott, Susan E. Watt

**Affiliations:** School of Psychology, University of New England, Armidale, NSW, Australia

**Keywords:** non-verbal accent, stereotypes, enculturation, thin slices of behavior, impression formation

## Abstract

Historically, racial appearance has been a common source of information upon which we categorize others, as have verbal accents. Enculturated *non-verbal* accents which are detected in facial expressions of emotion, hairstyle, and everyday behaviors, have also been found to exist. We investigated the effects of non-verbal accent on categorization and stereotyping when people are exposed to thin slices of behavior. The effects of racial essentialism, which inclines people to categorize and assess others by race, were also tested. In three studies, Australian participants were shown short, muted videos of target individuals performing everyday behaviors. The targets were of a minority (Asian) racial appearance, but half had been interracially adopted as babies and grew up in the Australian mainstream. The other half were foreign nationals who grew up in Asia. In Studies 1 and 2, Australian participants rated each target as Australian or foreign. In both studies, they correctly identified the targets at above chance levels. In Study 3, participants rated the targets on Australian and Asian stereotype traits. They were not told that some targets were Australian and some were foreign, but they nonetheless rated the congruent stereotypes more strongly. Lay theory of race moderated the effect of non-verbal accent, with a weaker effect among participants who endorsed racial essentialism. These preliminary findings reveal subtle effects of non-verbal accent as a cue to cultural group membership and invite further work into the effects of non-verbal accent on person perception and categorization processes.

## Introduction

Majority populations in multicultural societies generally consider it desirable that immigrants integrate, which involves interacting with and adopting aspects of the host culture while maintaining their culture of origin (Berry, 2006). They hold a more positive perception of individuals and immigrant groups who integrate, and this outcome seems to be regardless of whether the racial appearance of an immigrant is the same or different to the host majority (Van Oudenhoven et al., 1998; Maisonneuve and Teste, 2007; Alcott and Watt, 2017).

The concept of “race” is contentious. Historically it has referred to the division of humanity into groups that reflect an inherited biological foundation and is manifested in physical phenotypes, such as skin color, eye shape, hair texture, and bone structure. While these physical phenotypes are used as markers for categorization, the extent of racial essentialism regarding abilities, character, or behavior has been debunked on scientific and evolutionary grounds (Fishbein, 1996; Kurzban et al., 2001). Despite this, people all over the world categorize themselves and each other according to “race,” and it continues to be a salient factor in the organization of people's social worlds (Gossett, 1997; Celious and Oyserman, 2001). In the current research, we conceptualize “race” as “racial appearance,” referring to the physical phenotypes mentioned. Social perception is essentially categorical (Spears and Haslam, 1997), and for immigrants of minority racial appearance around the world, racial categorization can have substantial effects on social and national belonging. Racial appearance has often been relied upon as a cue to nationality. For example, studies conducted in the United States of America (USA) have shown that Americans of Asian descent are often labeled as “foreigners” rather than as Americans (Tuan, 1998; Devos and Banaji, 2005), and issues around identity and belongingness are frequent.

Racial appearance, or at least visible minority status, can be an obstacle to full participation in the majority culture for a variety of reasons and can create sensitivity to exclusion (Tafarodi et al., 2002). Research performed in Canada by Tafarodi et al. found that priming subjects of a racial minority (Chinese) for self-awareness of their physical appearance and racial minority status produced a “compensatory conformity” effect in the subjects. The compensatory conformity effect was expressed as a stronger alignment with majority group attitudes compared to those who did not have an awareness of their physical appearance heightened. Tafarodi et al. interpreted this as an effort to ensure inclusion and belonging with the majority population by individuals who did not want to be “ethnified” by the majority group members based on physical appearance. However, this is difficult if one of the conditions of being included in the majority population depends on being of the same racial appearance. For instance, France does not officially acknowledge race or ethnicity, as just being French is seen to be the most important identification (Beaman, 2018). However, it seems that having a white racial appearance is unofficially synonymous with being included as French by the majority population (Beaman, 2018). Many individuals (particularly North African second-generation immigrants) do not feel accepted as French, even though they were born there, because they are not seen to “look French” (Simon, 2012). In Australia, research has also demonstrated that being white is more readily associated with the concept of being Australian than is being Indigenous Australian (Sibley and Barlow, 2009).

### Minority Racial Appearance and Majority Enculturation

The current research investigated the attitudes and perceptions of an Australian majority population toward individuals who have a minority racial appearance but who are fully enculturated into the dominant mainstream culture. One example of this is generations “deep” immigrants who may no longer identify with the culture of their ancestors' country of origin, such as fifth or sixth generation Chinese Australians. Another example is people who are interracially adopted. Usually, people who are adopted into Australia from other countries are adopted and raised in white-Anglo homes. They may have little exposure to the culture from their country of birth and become fully enculturated into Australia's dominant mainstream culture. However, does their racial appearance preclude them from being included by the national majority members as a cultural ingroup? Are they destined to be perceived by the dominant majority as “not quite Australian?” It is important to investigate this question as the answer has ramifications not only for the lives of individuals who are interracially adopted and are in this situation but also for long-term immigrants who identify with the mainstream or dominant culture. The perceptions and attitudes of the dominant majority population toward minority racial groups influence the groups' inclusion and sense of belonging in the broader society.

### Non-verbal Accent as a Cue for Categorization

Understanding the effects of categorization on people who have a minority racial appearance and investigating how they are perceived and included by their compatriots is an interesting question to ask in light of the continuum model of categorization (Fiske and Neuberg, 1990). Among other things, this model proposes that sex, age, and race are “privileged” categories that mark social group membership. They are privileged because they are prominent, visual, and can be easily and immediately applied to most people we encounter. We, therefore, can instantaneously categorize others according to these three cues. There is evidence that racial categories are processed early in person perception. For example, American participants have been found to give preferential attention to race over the other two salient categories of sex and age (Ito and Urland, 2003), and studies have shown that race, as a social category, is processed in under 200 ms (Kubota and Ito, 2017). There is also the work by Greenwald and Banaji (1995) and the development of the Implicit Association Test (Greenwald et al., 1998), which demonstrates that people will react implicitly to racial stereotypes.

However, accents are also strong cues for social categorization (Ladegaard, 1998). While the usual understanding of accent is that it is an aspect of spoken language, research has also demonstrated the existence of non-verbal accents (Marsh et al., 2003, 2007). For example, emotional expression, a non-verbal behavior, is well-supported as being an effective channel of communicating meaning and has been characterized as being a universal language (Ekman, 1972, 1977), albeit a language that carries accents. Marsh et al. (2003) defined this non-verbal phenomenon as “non-verbal accent.” Like verbal accents, non-verbal accents arise in enculturation and signal one's cultural background, and it has been demonstrated that they reveal enough information that observers can identify the expresser's nationality. Research by Marsh et al. (2003) had American participants judge the nationality of people who were racially Japanese but had grown up either in Japan or the USA after looking at photographs that showed them expressing discrete emotions (e.g., sadness or anger). Participants correctly identified at above chance levels and with a large effect size, the nationality of the targets displaying these emotional expressions. They also found that the effect was much larger in photographs where emotions were being expressed rather than when targets had a neutral face. These findings indicate that expression of emotion conveys a non-verbal accent that can identify nationality or culture and that cultural differences are intensified when expressing emotions.

Recent research by Matsumoto and Hwang (2018) replicated Marsh et al.'s (2003) study to further investigate non-verbal accent, with a view to isolate cues that participants use to detect nationality. They used the same stimulus photos as Marsh et al. but manipulated the stimuli by switching hairstyles. While Marsh et al. concluded that facial expression of emotion was responsible for the results, Matsumoto et al. concluded that hairstyle differences contributed to differences in detecting nationality, especially in the judgment of Japanese nationals. However, in another study conducted by Marsh et al. (2007) using different stimulus photos (of white Americans and Australians), even after removing the targets' hairline and hair, participants could still correctly judge nationality at above chance levels, and more so when the targets expressed emotion on their faces.

Hamamura and Wai Li (2012) found that Hong Kong participants could detect whether or not a Hong Kong target identified with Western culture (as is commonly the case in Hong Kong) by observing muted 60-s videos of seated targets as they responded to questions asked by an off-camera interviewer. Targets were asked about their stress levels, how they managed their stress, and what things they hated or disliked. Questions like these with affective content may elicit emotional responses, and the detection of cultural influence may be due to non-verbal accents in facial emotional expression. The researchers recognized this and, to isolate cues, removed emotional expression in a subsequent study by using neutral photos of the target's head. They then took it a step further, removing the targets' hair, showing just the face with the hair removed. After removing the hair, the targets' cultural identification was no longer perceived. The authors concluded that hairstyles play a role in conveying cues regarding Western cultural identification among Hong Kongers. Thus, there is evidence for both hairstyles and facial expressions of emotion contributing to non-verbal accent.

Physical behaviors such as walking and waving have also been found to convey detectable cultural influences and can be considered to be part of non-verbal accent. Marsh et al. (2007) showed American participants static photographs of American and Australian targets either with their arm raised, waving, or walking mid-stride. The targets wore hairnets to minimize differences in hairstyle. They found that participants correctly identified at above chance level which targets were Australian, and which were American, with a medium to large effect size. Furthermore, participants assigned a dominant (congruent) stereotype to the Americans and a likable (congruent) stereotype to the Australian targets, demonstrating that manner of behavior played a role in determining cultural group membership.

While non-verbal accent was originally conceptualized as the subtle cultural differences evident in facial expressions of emotion (Marsh et al., 2003), the research described above extended this to include hairstyle and physical postures. We suggest that there are likely to be many more such cues, such as subtleties in the way one wears clothes, the way one walks, or how animated a person is during speech. Under controlled conditions, a single cue has been found to be sufficient to indicate one's enculturation. In the current research, we examined the effect of a combination of cues under naturalistic conditions. To capture the notion that non-verbal accent may be expressed by many features, the current research defined non-verbal accent as *the sum total of a person's enculturated, physical cues to their cultural background*. An important aspect of this definition is that non-verbal accent, just like verbal accent, arises through enculturation, that is, it naturally arises through a person's immersion in a particular culture.

### The Current Research

Most of the research into non-verbal accents has used highly controlled stimuli in laboratory settings. Marsh et al. (2003) suggested it would be worthwhile for future work to explore how people use the information from non-verbal accents in naturalistic settings. The present research aimed to extend our understanding of non-verbal accent to the cues conveyed in brief observations of everyday behaviors, like seeing others walking or running along a street, or having a conversation which can be observed but not heard, such as when in a café and observing a stranger across the room. The technique of *thin slices of behavior* offers an ideal method for mimicking naturalistic encounters. “Thin slices of behavior” refers to short glimpses of dynamic behavior, typically presented in short videos, that provide enough information for observers to form impressions of the targets being viewed (Ambady and Rosenthal, 1992).

Previous studies have been conducted on detecting the national identification of people with a minority racial appearance in the United States of America (e.g., Marsh et al., 2003) and in Canada (Bjornsdottir and Rule, 2020). However, those studies used photographs and focused on non-verbal accents in the facial display of emotion. The current research expanded from faces to thin slices of behavior, which incorporate everyday movement and behaviors in a naturalistic setting, and tested the impression formed by seeing the whole person, as is more commonly observed in everyday situations. Another goal, using these stimuli, was to investigate non-verbal accent as a cue to national categorization and stereotyping when the target person is of a minority racial appearance, using thin slices of behavior in a naturalistic setting. We wanted to know if people who have a minority racial appearance but local enculturation would be categorized and stereotyped by Australian citizens as fellow nationals. People who are interracially adopted and who have a minority racial appearance are a strong exemplar of this situation and were the focus of this research. Conversely, we also asked if people who have a minority racial appearance, but foreign enculturation, would be categorized and stereotyped as foreign, at above chance levels.

Three studies were conducted in Australia, a country built on immigration, with 28% of its current population born overseas. While Australia is a multicultural and multiracial nation, the dominant majority population is racially white with an Anglo/European ethnic heritage. Australia has a small number of interracially adopted people (just over 4,500 in the last two decades; Australian Institute of Health Welfare, 2018). Nevertheless, intercountry adoption is intricately connected with society's ideas about race, culture, ethnicity, kinship, and belonging to family and nation (Volkman, 2005). Australia provides a natural context for examining if and how people who have been interracially adopted, and how people of racial minorities generally, are identified as members of the larger mainstream national/cultural group.

#### Lay Theory of Race

Individual differences in attitudes toward race are likely to affect social categorizations (No et al., 2008). This paper uses the lay theory of race to explain how people understand the concept of race and how it may affect their judgments of others. According to the lay theory of race, people endorse either “racial essentialism” or “social constructionism” lay theories. People who endorse social constructionism view race and its effects as a social construction that is malleable and context-driven (No et al., 2008). Therefore, based on non-verbal accent, people who endorse social constructionism might easily categorize someone with a minority racial appearance as having majority enculturation. On the other hand, people who endorse racial essentialism believe racial groups have inherent natures that are biologically based, innate and immutable (Haslam et al., 2000; Prentice and Miller, 2007). They also perceive well-defined boundaries that are both social and physical to delineate members of different racial groups (Chen and Hamilton, 2012), and believe that “race” is highly informative of a person's physical and psychological characteristics (Rothbart and Taylor, 1992). Furthermore, research has found that people who endorse racial essentialism are more likely to categorize based on race (Chao et al., 2013). Therefore, an effect of non-verbal accent might not be present when people endorse racial essentialism, because they may not see past a minority racial appearance and not be sensitive to the non-verbal manifestations of mainstream enculturation.

The present studies aimed to put some well-known effects from the impression formation literature to the test in a dynamic environment, more akin to many real-world impression formation situations. Non-verbal accent was operationalized by developing a 60-s, silent video of each target person as they performed a set of everyday behaviors. The targets were people of the same minority East Asian racial appearance who had been adopted as babies and raised in Australia, or foreign nationals who had grown up in Asia and had been in Australia for <2 years. This time frame was used as a precaution in case non-verbal accent changes upon immersion in a new culture.

Non-verbal accent has been shown in previous studies to reflect one's enculturation or cultural identification (in the case of Hamamura and Wai Li, 2012). We, therefore, expected that targets who were raised in Australia would present an Australian non-verbal accent, and those raised in Asia would present a foreign non-verbal accent. This was tested in Study 1, which investigated whether participants would correctly identify people who had been adopted interracially into Australia and were fully enculturated Australians as being Australian, and those who were foreign nationals as not Australian. We predicted that they would be able to do this at above chance levels through the influence of non-verbal accent. We also probed the reasons why participants decided a target was Australian, asking them a free response question after they had made their choice, to discover whether participants were aware of any aspect of non-verbal accent that influenced their decision. A second study explored the effect of lay theory of race. We predicted that participants who scored high in racial essentialism would be less accurate when identifying nationality than participants who scored high in social constructionism. A final study investigated the influence of non-verbal accents by asking participants to rate each target against a mixed set of Australian and Asian stereotype traits. We predicted that the targets who were interracially adopted would be allocated higher scores than foreign targets on Australian stereotypes than Asian stereotypes. We further predicted that the effect would be moderated by lay theory of race as people who endorsed racial essentialism would be more influenced by racial appearance (and less influenced by non-verbal accents) in their stereotyping than those who endorsed social constructionism.

## Study 1

### Method

#### Participants

Two hundred and five participants aged between 18 and 83 years (*M* = 47, *SD* = *17.2*) were recruited from Qualtrics online panels. Forty-eight percent were males, and all participants were Australian citizens, with 78% born in Australia. Those not born in Australia had lived in Australia for an average of 33 years (minimum = 3 years, maximum = 70 years). Two participants did not complete the study and were removed from the dataset. The University's Human Research Ethics Committee did not permit us to ask about participants' racial background. However, an earlier study using the same recruitment method and on a similar topic (Alcott and Watt, 2017) produced a sample with 90% Caucasian/Anglo/European ancestry and 6% Asian ancestry. The remaining 4% had ancestry from other parts of the world.

#### Design

A single factor experimental design was used, where targets' enculturation (Australian, foreign) and, therefore, non-verbal accent, was a within-subjects factor. The dependent variable was the identification of the target's nationality. The stimuli comprised six standardized 60-s silent videos, with one target person shown in each video as they performed several everyday behaviors. The targets were three interracial adoptees and three foreign nationals. All targets were male and were matched for age (1 × early 20's, 1 × late 20's—early 30's, 1 × 40's), general appearance, and the wearing of glasses (1 × glasses in each group). The targets are shown in Table 1 below. Locating male interracial adoptees from East Asia who were willing to be filmed and then matching them with recent arrivals in Australia according to the criteria of age, general appearance and glasses-wearing was not an easy task, and within the constraints of this project, it was not possible to conduct further filming. Viewing six stimuli was a number that seemed acceptable in terms of participant concentration and fatigue on watching and rating the six videos and providing an open-ended response for each.

**Table 1:** Target stimuli.

**Foreign nationals**			
Target individual	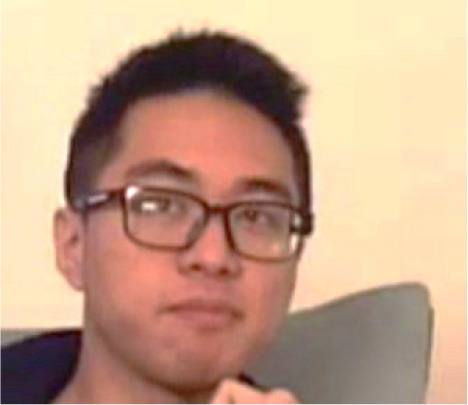	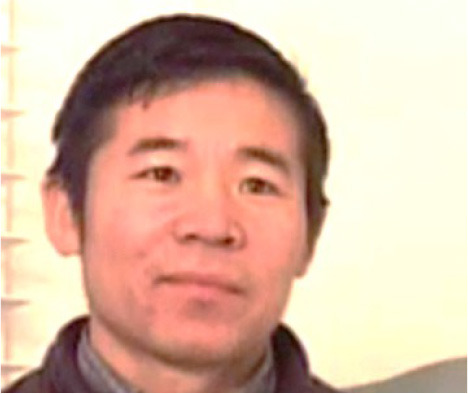	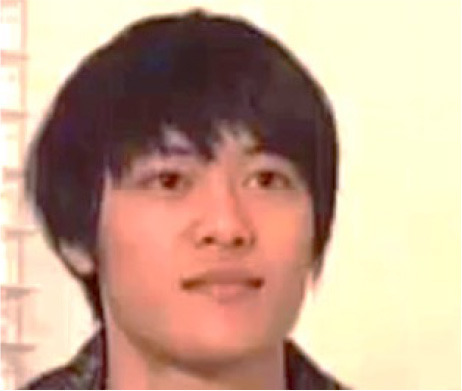
	Man A	Man B	Man C
Country of birth	China	Mongolia	China
Time in Australia	5 months	2 years	8 months
**Interracially adopted Australian Nationals**			
Target individual	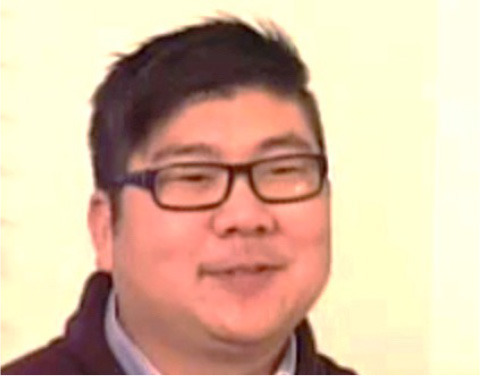	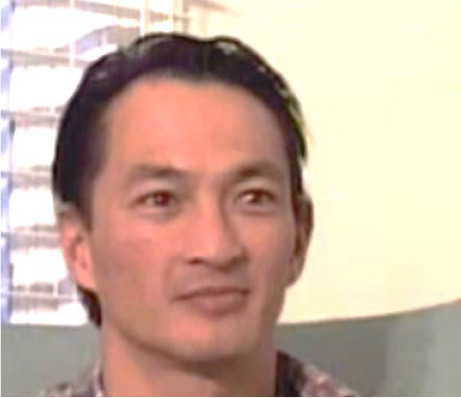	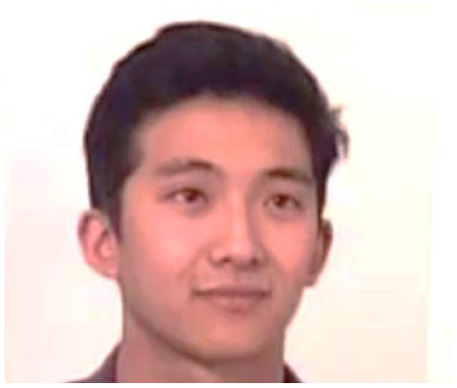
	Man D	Man E	Man F
Country of birth	South Korea	Vietnam	South Korea
Time in Australia	From a baby	From a baby	From a baby


Previous research by Marsh et al. (2003) and Marsh et al. (2007), which used unbiased hit rates, found effects of non-verbal accent with a medium effect size, and research by Hamamura and Wai Li (2012) using silent videos of people talking about an emotional topic revealed medium to large effect sizes. However, as thin slices of behavior had not previously been used in this way for non-verbal accents research, we computed the sample size on an expected small effect size *(f* = 0.10) and power of 0.90. A G^*^Power (Faul et al., 2007) analysis revealed that 179 participants were required for the 2-way within-subjects analysis required to compare hit rates with chance responding for foreign and Australian targets (see “Data analysis”).

#### Stimuli—Films of Targets Displaying Non-verbal Accents

We created six videos that each showed an individual of Asian racial appearance. Three were interracial adoptees (two from South Korea and one from Vietnam) who had grown up in Australia and were Australian nationals. They were located *via* adoption websites and through contacts of the authors and were aged between 20 and 45 years (see Table 1). The other three were foreign nationals who were temporarily visiting Australia. They were recruited from English as a Second Language Schools in Sydney, were between 20 and 45 years of age, and were from China and Mongolia (see Table 1). They had been in Australia for <2 years. Using a cut-off of 2 years was a precautionary decision. No evidence was available stating when non-verbal accents begin to change on moving countries, and so a conservative estimate of 2 years was used in case people who had been in-country for longer would be poor exemplars of the foreign non-verbal accent. One target who had been in Australia for 5 years was accidentally included in the stimulus set. Data relating to that target were not included in the final analyses.

All targets were paid for their time and consented to their images being used for research purposes. Before the filming, the targets were not given information about the research objectives, except that it was investigating race relations in Australian society. They were fully debriefed afterward. Based on research by Eagly and Kite (1987) which found that men are perceived to resemble stereotypes of their nationalities more than women, and to reduce any effects of gender, only male targets were used in the scenarios.

All attempts were made to make the targets relaxed and at ease during filming. For example, they were given time to get acquainted with the researcher and assistants, were offered refreshments, and given time to get comfortable before filming began. The foreign nationals could speak English and were selected by their teacher for competence and ease while communicating in English. To ensure their ease during the filming, they were given the translated questions beforehand, so they could prepare and feel comfortable in the interview.

##### Video Content

Filming took place over six separate sessions (one session for each subject) and was in the same location each time. Each subject walked down the same suburban footpath, waited while using a mobile phone in the same spot each time, ran down the same stretch of the footpath, and finally was seated, having a conversation with an off-camera interviewer in the same chair/lighting, etc., of the same room as they answered simple non-affective questions about their lives (e.g., “Where do you live?” “Do you play any sports?” “What are your hobbies?”).

The soundtrack of the conversation was entirely removed; only ambient sounds could be heard. The subjects were asked to wear their everyday casual clothes and were advised they were not required to “act” but just be themselves. Finally, the films were all edited in the same manner, with the cuts made at the same time points, so far as possible. The final length of each film was 60 seconds. The films were presented to participants in random order.

#### Measures

##### Identifying Nationality

Participants viewed six separate 60-s videos, where each video showed a target person performing several everyday behaviors. Following each video, they were asked, “*Please indicate if you agree with this statement: ‘The man in the film is an Australian (has grown up in Australia)'* (Yes/No).”

##### Open Response

After identifying the nationality of each target, participants were offered the option of explaining their decision with an open response question: “Can you say what made you answer yes or no?” Several themes emerged from the responses, based on references to movement, demeanor, and attitude, clothing, and hair, or a more general description of the target. Three independent judges allocated the responses into the thematic categories, and Krippendorff's alpha was calculated to assess agreement between the judges, using the Kalpha macro (Hayes and Krippendorff, 2007).

##### Previous Knowledge Check

We checked whether participants knew any of the men in the videos. The question asked, “*Do you know any of the men in any of the videos?”* None of the participants knew any of the targets.

#### Procedure

The experiment was presented *via* an anonymous online survey. Participants were told that it was an investigation into how much information is needed to form first impressions. They first completed a demographics questionnaire (citizenship, age, gender, born in Australia, years lived in Australia). There was then a brief familiarization trial in which they were shown 20 s of the videos and were asked to rate, as a filler task and to support the cover story, the targets on the dimensions of warmth and competence (Fiske et al., 2002). This is commonly done in such judgment studies (e.g., Matsumoto, 1993; Marsh et al., 2003, 2007) so that participants are familiar with the format of the stimuli, allowing them to adapt to the situation, which can enhance the quality of data (Barley, 2011).

After the familiarization trial, they were told that some of the targets had grown up in Australia and were Australian nationals, while some were foreign nationals who had not been in Australia for very long. This ensured participants understood that each target had a chance of being an Australian national or not, which limits test bias (Marsh et al., 2003). Participants then watched the full 60-s videos and judged the national identity of each target, stating who was Australian (has grown up in Australia) and who was not.

#### Data Analysis

No analyses were conducted before the data gathering was completed. Participants' judgments of the targets' national identities were classified as correct or incorrect, and then unbiased hit rates, *Hu*, were computed using the method developed by Wagner (1993). *Hu* takes into account whether participants or stimuli might be biased toward a particular response. It takes “simultaneous account of both stimulus and judge performance” (Wagner, 1993, p. 16) by combining the conditional probability of a stimulus being correctly identified and a response being correctly used (please see Wagner, 1993, p. 17). It is then compared with chance performance to determine if the unbiased hit rate is significantly different to chance. In this way, *Hu* accounts for whether stimuli in one category might produce bias toward one type of response over another, and whether some participants might be biased toward a particular response. It combines these into a measure of accuracy ranging from zero to +1. If a participant responded “Australian” to all targets, the *Hu* score for the foreign targets would be 0 as the participant was wrong on each occasion. Even though all ratings of Australians would be correct, the *Hu* score for the Australian stimuli would be 0.5, not 1, because the calculation of *Hu* takes into account the over-use of the Australian category. Perfect accuracy in categorizing the Australian and foreign targets would yield a *Hu* score of 1 for both categories, and perfect inaccuracy would yield a *Hu* score of 0 for both categories. Using Wagner's method, the *Hu* score can be compared with chance performance (*pc*) of each stimulus/judgment combination, which is also rated from 0 to +1. The comparison of *Hu* and *pc* is assessed *via* paired *t*-tests, or in a more complex design, repeated measures ANOVA or MANOVA. Because the current design had two types of targets, foreign and Australian, we conducted a 2 (hit rate: *Hu, pc*) x 2 (non-verbal accent: foreign, Australian) repeated measures ANOVA using SPSS version 26. The distributions of all variables were checked before analysis.

### Results

#### Identifying Nationality

The repeated measures ANOVA revealed a significant effect of hit rate with a large effect size, *F*_(1,201)_ = 58.56, *p* < 0.001, partial η^2^ = 0.226. Unbiased hit rate, *Hu* (*M* = *0.3*7, *SE* = 0.015, CI_lower_ = 0.34, CI_Upper_ = 0.40) was significantly higher than *pc* (*M* = *0.2*5, *SE* = 0.000, CI_lower_ = 0.25, CI_Upper_ = 0.25). There was no interaction between hit rate and target culture, *F*_(1,201)_ = 0.49, *p* = 0.48, partial η^2^ = 0.002. Thus, participants performed at above chance accuracy when classifying both foreign and Australian targets.

#### Open Responses

The open responses were analyzed to shed light on how participants correctly identified the Australian targets as Australian. Participants were invited to explain their decision following each target. Out of 361 correct identifications of Australian targets, 299 open responses were provided. These were analyzed to identify if participants were aware of relying on any particular aspects of non-verbal accent when deciding that a target was Australian. Comments that made no sense or were irrelevant were removed (*N* = 26), leaving 273 responses for coding. Preliminary coding revealed five thematic categories (see Table 2). Three independent judges were then recruited to rate the comments against the thematic categories, and Krippendorff's alpha was computed to measure agreement between the judges. The result of α = 0.86 indicates acceptable interrater reliability (Krippendorff, 2010). Only the comments with 100% inter-rater agreement were included in the percentages for each category, as presented in Table 2. Examples of the comments are included.

**Table 2:** Thematic categories, percentages, and examples.

**Theme**	**Percent (*N* = 273)**	**Comment examples**
Confident/comfortable	21%	“*He strikes me as quite confident…”; “Relaxed and easy confident manner”; “Comfortable confidence.”*
Laid Back/easy going/casual	19.5%	“*His casual approach”; “He appeared laid back enough to be an Aussie”; “A very relaxed and laid back individual.”*
Movement style	15%	“*He just ambled along.”; “How he walks and runs—very relaxed”; “Swaggering and relaxed walk”; “How he runs and his casual walk style.”*
Dress & hair	10%	“*.the way he is dressed seems Australian”; “His hairstyle, outfit and movement”; “He wears Aussie clothes”*
Don't know/just an impression	34.5%	“*His demeanor”; “Seems to be a typical Aussie”; “Just a feeling”; “Looks that way is all I can say”; “Just a gut feeling”; “Very typical Australian”; “Just do.”*

### Discussion

This study tested if non-verbal accent is a discernible marker of enculturation and nationality when presented briefly in thin-slices of spontaneous behavior. We hypothesized that Australian participants would be able to identify people who had grown up in Australia as Australian by briefly observing thin slices of their behavior, which potentially conveyed non-verbal accents. The targets were people of a minority Asian racial appearance who had either been interracially adopted into Australia or were recent arrivals to Australia. Race commonly has a significant influence on categorization, but if race were the only influence, we would expect no difference between the two groups of targets because race was held constant across the conditions. The results supported the hypothesis because, on average, participants correctly identified the Australians at above chance levels. That participants could identify the targets' nationality based on their non-verbal accent builds upon previous research which has found that non-verbal accent in emotional expression, communicative behaviors such as waving, and instrumental behaviors such as walking is sufficient for participants to correctly infer cultural and national differences (Marsh et al., 2003, 2007). While the targets in the current study did not demonstrate specific, discrete emotional expressions (e.g., happiness, sadness, anger) or overt communicative behaviors like waving, they did display ordinary behaviors that one may observe another performing in everyday life. The effect sizes were small to medium, and the results indicate that people who have been adopted interracially into Australia and who have a minority racial appearance display non-verbal accents that signal their national belonging.

While the free responses supported that the participants based their decisions on various elements of non-verbal accent, the largest percentage tended to see the overall effect of nonverbal accent rather than the components. This reflects a comment of Marsh et al. (2007) that participants in their study responded to a gestalt impression—meaning they responded to the overall impression, rather than the individual components of the target.

There are further questions about the moderators of the effect. Namely, is non-verbal accent a useful source of information if one endorses an essentialist lay theory of race? Racial essentialism purports that race is inevitably associated with a person's traits and abilities; that race is biologically based and genetically determines behavior, justifying endorsements of racial stereotypes (Jayaratne et al., 2006). Therefore, a person who endorses racial essentialism may have difficulty in identifying ingroup enculturation in someone whose racial appearance represents “outgroup.” Because the two lay theories of race (essentialism and social constructionism) understand race differently, in Study 2, we predicted that participants who endorsed social constructionism would be open and sensitive to the non-verbal accents of the individuals who have been interracially adopted. They would, therefore, be able to identify them as Australian at above chance level. On the other hand, the perceptions of participants who endorsed racial essentialism would be dominated by the target individuals' racial appearance and would therefore not respond to the effect of the individual's non-verbal accent.

## Study 2

### Method

#### Participants

Two hundred and twelve participants were recruited from Qualtrics, an online participant panel. There were equal numbers of male and female participants, with a mean age of 47 years, *SD* = *17.2* (minimum = 18 years, maximum = 86 years). All were Australian citizens, and almost 80% were born in Australia. The remaining 20% who were not born in Australia had lived an average of 32 years in the country (ranging from 5 to 68 years).

#### Design

A single factor experimental design was used, where targets' culture (Australian, foreign) was a within-subjects factor. Lay theory of race was measured as a continuous moderator. The dependent variable was the identification of Australian nationality.

Study 1 revealed a strong effect of non-verbal accent, but we anticipated a small interaction effect of lay theory of race. A G^*^Power analysis computed on an expected small effect size *(f* = 0.10) with power of 0.90 indicated that 180 participants were required.

#### Stimuli

The stimuli were the videos that were used in Study 1. They were presented in random order.

#### Measures

The same identification of nationality was used as in Study 1. Study 2 also included a measure of lay theory of race.

##### Lay Theory of Race Scale

The Lay Theory of Race Scale (No et al., 2008) was presented to participants as investigating how people understand the notion of race. The scale consists of eight items that determine whether a respondent endorses racial essentialism or social constructionism. Four items measure racial essentialism (e.g., “*What a person is like (e.g., his or her abilities or traits) is deeply ingrained in his or her race. It cannot be changed much.”)* and four items measure social constructionism (e.g., “*Racial groups do not have inherent biological bases, and thus can be changed.”)*. Participants were asked to rate each item on a scale from 1 (strongly disagree) to 6 (strongly agree). We reverse-scored the social constructionism items so that high scores reflected an endorsement of racial essentialism and low scores, social constructionism. This procedure, presentation, and scoring of the lay theory of race measure were consistent with previous research (No et al., 2008) and simultaneously measured participants' lay theory of race and primed whichever lay theory the participants endorsed before they completed the next task. Cronbach's alpha for the scale was 0.63, which was a little lower than No et al.'s (2008) alpha of 0.76.

#### Procedure

The procedure was the same as in Study 1, except participants also completed the lay theory of race scale before viewing the videos.

#### Data Analysis

No analyses were conducted before the data gathering was completed. Participants' judgments of the targets' national identities were classified as correct or incorrect, and then unbiased hit rates, *Hu*, and chance performance, *pc*, were computed in the same way as in Study 1. To identify whether participants were able to identify nationality at above chance level, *Hu* was compared with *pc*. As the moderator, lay theory of race, was a continuous between-subjects variable, a linear mixed model was computed using the GAMLj module in Jamovi (Version 2.0.6.; Gallucci, 2019). Participant was added as a random effect, and the fixed effects were target culture (foreign, Australian), hit rate (*Hu, pc*), and lay theory of race. The interactions of target and lay theory of race with hit rate were assessed, as was the three-way interaction of target, lay theory of race, and hit rate. Jamovi computes degrees of freedom for *t*-tests and *F*-tests of the main model in linear mixed models using the Satterthwaite method. *F*-tests for simple effects use the Kenward-Roger method to compute degrees of freedom.

### Results

Consistent with Study 1, the fixed effect omnibus tests revealed a significant effect of hit rate [*F*_(1,588)_ = 85.51, *p* < 0.001] such that *Hu* (*M* = 0.37, *SE* = 0.01, CI_lower_ = 0.35, CI_upper_ = 0.39) was significantly higher than *pc* (*M* = 0.25, *SE* = 0.01, CI_lower_ = 0.23, CI_upper_ = 0.27). There was no interaction of lay theory of race and hit rate, *F*_(1,588)_ = 0.93, *p* = 0.337. Therefore, participants were able to identify nationality at above chance levels, regardless of their lay theory of race. There was no interaction of target culture with hit rate [*F*_(1,588)_ = 0.00001, *p* = 0.997], nor was there a three-way interaction of target culture x hit rate x lay theory of race [*F*_(1,588)_ = 0.00003, *p* = 0.996).

### Discussion

The hypothesis for this study predicted that participants who endorsed racial essentialism would not be able to discern who was Australian because they would assess the targets on racial appearance and not be sensitive to the non-verbal accent of the target individuals. This hypothesis was not supported. The results demonstrated no significant difference between participants who endorsed racial essentialism and social constructionism. Both groups were able to detect nationality at above chance levels based on non-verbal accent, and there was no significant difference in their accuracy.

In a country such as Australia, which has a population of people from diverse ancestries, racial appearance is not a barrier to formal national citizenship. Someone who endorses racial essentialism is just as likely as someone who endorses social constructionism to understand that a person can migrate from another country and be an Australian national while having an Asian racial appearance (or any racial appearance).

So, while Australian participants may attribute ingroup nationality to an individual who has a minority racial appearance, they may not attribute typical Australian traits to these individuals. That is, they may not include them in the “stereotypical cultural ingroup.”

Hamamura and Wai Li (2012) and Marsh et al. (2007) proposed that the accuracy of nationality judgments based on non-verbal cues may depend on the stereotypes that observers hold about the members of the national group. In our first study (Study 1), when participants were asked why they deemed the target to be either Australian or a foreign national, we found their comments hinted at relying on commonly known Australian stereotypes (e.g., laid back, easy-going) to make their decision.

Study 3 investigated the role of stereotypes. It had two goals. The first goal was to remove the task of identifying nationality and instead test if participants could discern people who are enculturated Australians by asking them to attribute Australian or Asian stereotypes to the targets. The second goal was to see if participants who endorsed racial essentialism would attribute Australian stereotype traits to Australian people who have a minority Asian racial appearance (in this case, people who have been interracially adopted).

We hypothesized that non-verbal accent would be a cue upon which participants base social judgments. This would be demonstrated if participants attributed congruent (Australian) stereotypes to Australian targets who have an Asian racial appearance, rather than attributing incongruent (Asian) stereotypes to this group, and vice-versa. Therefore, an interaction between target culture and stereotype group was expected. We also hypothesized that participants who endorsed social constructionism would more readily apply congruent (Australian) stereotypes to the Australian targets than those who endorsed racial essentialism. Racial essentialism inclines people to categorize by race (Chao et al., 2013), so these participants would, therefore, allocate lower scores on Australian stereotypes to the Australian and foreign targets because of their Asian racial appearance. In this way, lay theory of race was expected to modify the interaction between target and stereotype, such that a three-way interaction would be present.

## Study 3

### Method

#### Participants

Two hundred and eight Australian citizens were recruited as participants *via* Qualtrics online panel (52% female, 48% male). The mean age was 47 years, *SD* = *18.0* (minimum = 18 years, maximum = 86 years). Seventy-nine percent were born in Australia. The remaining 21% who were not born in Australia had lived in Australia an average of 34 years (ranging from 2 to 68 years). In this respect, the sample was representative of Australia's population, where approximately 25% are born overseas (Australian Bureau of Statistics, 2016).

#### Design

A 2 (non-verbal accent: Australian, foreign) x 2 (stereotype: Australian, Asian) mixed experimental design was used, together with a continuous moderator, lay theory of race. The dependent variable was endorsement of Australian and Asian stereotype traits. Like Study 2, we estimated that 180 participants were needed to detect an effect size of *f* = 0.1 at power = 0.90.

#### Stimuli

The stimuli were the videos that were used in Study 1 and Study 2. They were presented in random order.

#### Measures

##### Lay Theory of Race Scale

The Lay Theory of Race Scale (No et al., 2008) was the same as used in Study 2. The items were presented in random order. Cronbach's alpha for this scale was 0.59.

##### Stereotypes

After each video, participants were shown a list of 10 traits and were asked to rate from 1 (“definitely not”) to 5 (“definitely yes”) how much each trait applied to the person in the video. The traits included five commonly held Australian stereotypes (down to earth, good sense of humor, friendly, laid back, outgoing) which were chosen from a preliminary study conducted by the authors (Alcott, 2019) as well from other studies examining Australian stereotypes (Haslam et al., 2000; Leeson, 2016). There were also five commonly held Asian stereotype traits (courteous, quiet, sincere, shy, traditional) which were chosen from various studies that examined consensual Australian stereotypes of Asian Australians as well as commonly held stereotypes of Asian Americans (Karlins et al., 1969; Borresen, 1982; Jackson et al., 1996; Johnson et al., 2005). The scores for the Australian stereotypes were calculated separately for the Australian and Asian targets. In this way, a total Australian stereotype endorsement and a total Asian stereotype endorsement was calculated for each group. The possible range of scores for Australian and Asian stereotypes for each group was 15–75.

##### Previous Knowledge Check

The same check as in Studies 1 and 2 was included at the end of the survey, in case participants knew any of the men in the videos.

#### Procedure

Participants completed an anonymous survey online. The study was presented as an investigation into how much information we need to form a first impression of others. Participants completed the demographic questions (Australian citizenship, gender, age, born in Australia, years lived in Australia) followed by the Lay Theory of Race scale (No et al., 2008). They then watched the 60-s videos of the targets and, after each one, scored the target on the list of descriptors provided. Due to the lengthy nature of the survey in this study, and to avoid participant fatigue, a familiarization task was not included.

#### Data Analysis

No analyses were conducted before the data gathering was completed. A linear mixed model was computed using the GAMLj module in Jamovi (Version 2.0.6.; Gallucci, 2019). Participant was added as a random effect, and the fixed effects were target (foreign, Australian), stereotype (Asian, Australian), and lay theory of race. The full factorial model was assessed.

### Results

The first hypothesis predicted an effect of non-verbal accent, such that Australian targets would be rated higher on the congruent (Australian) stereotypes than on the incongruent (Asian) stereotypes, and foreign targets would be rated higher on the congruent (Asian) stereotypes than the incongruent (Australian) stereotypes. The results supported this hypothesis, with a significant interaction between target and stereotype, *F*_(1,613)_ = 48.38, *p* < 0.001. When the target was foreign, the congruent Asian stereotype (*M* = 49.1, *SE* = 0.56, CI_lower_ = 48.0, CI_upper_ = 50.2) was rated higher than the incongruent Australian stereotype (*M* = 46.9, *SE* = 0.56, CI_lower_ = 45.8, CI_upper_ = 48.0), and when the target was Australian, the congruent Australian stereotype (*M* = 49.9, *SE* = 0.56, CI_lower_ = 48.8, CI_upper_ = 51.0) was rated higher than the incongruent Asian stereotype (M = 47.2, *SE* = 0.56, CI_lower_ = 46.1, CI_upper_ = 48.3).

**Figure 1:**
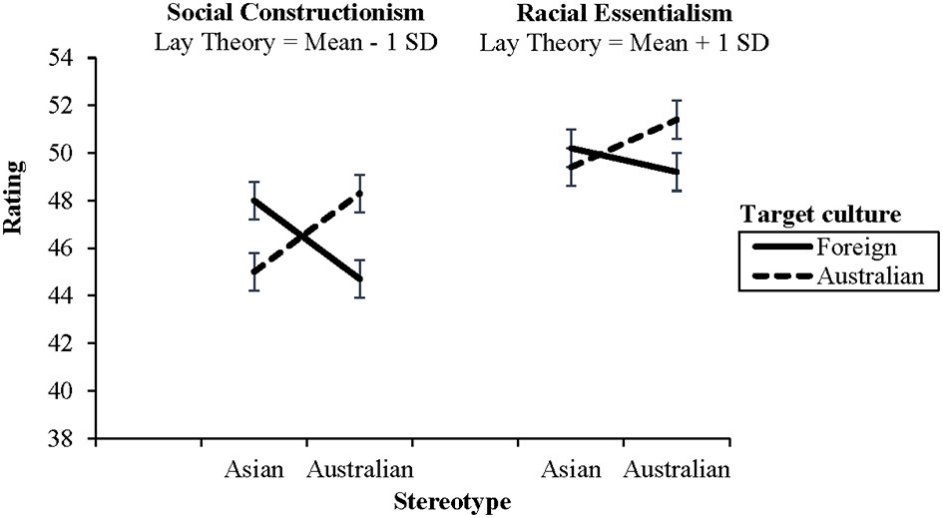
Ratings of Asian and Australian stereotypes as an effect of lay theory of race when target culture was foreign or Australian, computed at 1 sd above and below the mean for lay theory of race. Error bars are standard error.

The second hypothesis concerned lay theory of race, predicting that participants who endorsed social constructionism (low scores on lay theory scale) would more readily apply (congruent) Australian stereotypes to the Australian targets than those who endorsed racial essentialism (high scores on lay theory scale), resulting in more effect of non-verbal accent among those who were high in social constructionism. The results revealed a significant main effect of lay theory of race on stereotyping, *F*_(1,205)_ = 14.11, *p* < 0.001, such that high lay theory predicted higher stereotyping scores overall, with a medium effect size (*b* = 0.30). A significant three-way interaction was found between lay theory of race, target culture, and stereotype, *F*_(1,613)_ = 6.58, *p* = 0.01. As shown in Figure 1, the congruent stereotype was rated higher for both Asian and Australian stimuli, but this was more pronounced when lay theory of race was low (social constructionism).

To explore the effect size of the interaction of target culture and stereotype at high and low levels of lay theory of race, we divided the participants into high, medium, and low groups on lay theory of race using a tripartite split of this continuous variable. A 2 (target culture: foreign, Australian) x 2 (stereotype: Asian, Australian) repeated measures ANOVA was computed for the high lay theory group, and another was conducted for the low lay theory group. Supporting the second hypothesis, when lay theory of race was low (social constructionism), the interaction of target culture (*via* non-verbal accent) and stereotype was significant and with a strong effect size, *F*_(1,69)_ = 31.82, *p* < 0.001, partial η^2^ = 0.316, and when lay theory of race was high (racial essentialism), the interaction of target culture and stereotype remained significant, but with a much weaker effect size, *F*_(1,64)_ = 6.51, *p* = *0.0*13, partial η^2^ = 0.092.

### Discussion

We predicted that non-verbal accent, observed in thin slices of everyday behavior, would influence the allocation of national and racial stereotypes. We specifically hypothesized that participants would attribute Australian stereotypes to Australian interracially adopted targets (with Asian racial appearance) rather than attributing Asian stereotypes to this group, and vice-versa. This hypothesis was supported. Importantly, the participants were blind to the real purpose of the experiment. They were not informed that the traits represented national stereotypes, and they did not know that they were observing members of different national and cultural groups. They also did not know they were identifying compatriots and foreigners. Despite these conditions, participants still allocated Australian stereotypes to fellow Australians and Asian stereotypes to Asian nationals.

We also predicted that lay theory of race would influence how national ingroup stereotypes were attributed. The results showed an effect of non-verbal accent on stereotype ratings regardless of lay theory of race. However, this was more pronounced among those who endorsed social constructionism.

## General Discussion

The current research asked whether non-verbal accent is a cue to national identification when it is viewed as thin slices of behavior. Previous research has examined non-verbal accent in highly controlled conditions, usually varying one feature at a time. In contrast, we presented naturalistic stimuli that showed a person's physical presence as a whole, to determine whether non-verbal accent would affect categorization and stereotyping when a person forms first impressions on observing another person. After viewing the thin slices of behavior videos, Australian participants correctly identified targets of a minority racial appearance as Australian or foreign at above chance levels (Studies 1 and 2) and rated congruent stereotypes more highly than incongruent stereotypes (Study 3), even though they did not know that the traits were stereotype traits or that the targets represented two different nationalities. This further extends research that has been conducted to investigate non-verbal accents (Marsh et al., 2007; Hamamura and Wai Li, 2012; Matsumoto and Hwang, 2018), all of which have pointed to a role of stereotypes *via* non-verbal accent in discerning the cultural identification or nationality of target individuals.

We also wanted to understand the effect an individual's lay theory of race had on being able to identify Australians and foreign visitors. In Study 2, we predicted that endorsing a racial essentialist view would hinder this process, however this prediction was not supported. Although, in Study 3, there was an effect of lay theory of race. Australia is an “immigrant nation” and as such it is easily and readily understood that a person from any racial or cultural background could be an Australian citizen. So, endorsing racial essentialism would not be a hindrance to identifying people with a minority racial appearance as Australian. However, including targets of minority racial appearance into the “stereotypical cultural ingroup” may not be as easily done by people who endorse racial essentialism. Participants who endorsed social constructionism were slightly more adept at allocating congruent stereotypes to the two target groups which supported our prediction in Study 3.

Previous research has sought to separate and discern non-verbal accent cues that are used when judging another's national cultural group. The results have been mixed. For example, is it emotional expression (Marsh et al., 2003)? Is it communicative movements such as waving (Marsh et al., 2007)? Is it hairstyle (Hamamura and Wai Li, 2012; Matsumoto and Hwang, 2018)? Participants' comments in Study 1 shed some light on this. Hair and dress style were seldom mentioned, even though the targets wore their usual hair and clothes as we reasoned that these might naturally form part of non-verbal accent. A confident demeanor, behavioral style (casual, relaxed), and movement style (swaggering walk, running style) were commonly mentioned as reasons why respondents decided the target was Australian.

A large percentage of the open responses showed that participants could not articulate why they made their decision. This adds support to (Marsh et al.'s (2003)) suggestion on finding that participants did not seek out particular physical disparities in judging nationality, that participants may have been more attuned to a gestalt of differences, meaning that the whole conveys more than the individual parts. As the open responses indicate, some decisions on who is Australian were based on a nebulous “looked Australian” and “seemed Australian.”

One of our goals was to assess the influence of non-verbal accent in common everyday encounters, to add to the ecological validity of the research. That we obtained the current results from participants viewing such brief moments of non-verbal behavior is remarkable and begs the question, how far can we reduce the exposure to non-verbal accent before people can no longer perceive enculturation? Further research in this area would benefit from incrementally reducing the exposure time to discern this cut-off point.

### Limitations

The studies in this research all used the same stimulus set. As noted above, we were only able to locate and then match three targets from each group. Our results are, therefore, preliminary, and follow-up studies using different stimuli are required to solidify the conclusions.

Previous research has consistently shown effects of racial essentialism on categorization (Chao et al., 2013). However, the current results showed that people who endorse racial essentialism allocated stereotypes based on targets' enculturation (*via* non-verbal accent) rather than their minority appearance. These findings are curious and consideration around methods must be included. Although the focus of this study was on targets of minority racial appearance in Australian society, future research might include targets of varying racial groups. This may affect judgments of national identity and might also heighten the effect of racial essentialism as race becomes more salient by comparison.

The Lay Theory of Race scale showed reasonably low internal reliability in both studies, which could indicate the number of questions for each category was too low. In previous experiments on effects of lay theory of race, participants were primed for one theory or the other. No et al. (2008) argued that, depending on an individual's prior experience or social environment, lay theory of race might become more chronically accessible. They suggested it is also possible to increase the temporary accessibility of either social constructionism or racial essentialism by presenting participants with convincing evidence supporting that theory. This method has been shown to prime the corresponding theory in other research too, on implicit theories of morality (Hong et al., 2003) and gender (Coleman and Hong, 2008) and the propensity to categorize by race or theme (Chao et al., 2013). We did not prime our participants, nor did we embed the questions from the lay theory measure into a battery of other survey questions, as some previous research has done (Kung et al., 2018) to conceal the intention and minimize demand characteristics. To help ensure stronger endorsement of a lay theory of race, temporarily priming participants for either racial essentialism or social constructionism may be beneficial.

### Conclusion

The present research suggests that ethnic minority members' non-verbal accent, viewed as a combination of enculturated features, provides cues to their nationality. These are significant findings, particularly in the context of modern plural societies where one's racial appearance may not mean anything beyond a distant heritage. Members of racial minorities who are enculturated within the mainstream culture can be recognized as such, even from the moment of first impressions. This result was found in multiracial, multicultural Australia, where it is increasingly common for people with minority racial appearance to belong to the mainstream culture. Whether the same effect occurs in other countries is a matter for future research.

We focused specifically on interracially adopted individuals of minority racial appearance. However, the results could also reasonably generalize to immigrants more broadly and other racial groups. How long an immigrant retains their enculturated non-verbal accent is unknown; non-verbal accent could change quite quickly upon immigration, and as a precaution, we only included targets who had been in Australia <2 years. We found that ingroup non-verbal accent is a trigger for ingroup categorization and inclusion, but conversely, outgroup non-verbal accent could also trigger outgroup categorization, prejudice, and discrimination. Given the possibility that non-verbal accent is a barrier to intergroup acceptance, it would be useful to examine its duration upon migration.

Australian citizens accepted interracially adopted individuals as national ingroup members by identifying them as fellow Australian nationals and by applying Australian cultural stereotypes to them. This is a revelation to be pursued further for its implications on the inclusion of Australians of diverse racial backgrounds. There are many interracially adopted people and others who have immigrated to Australia, who “look Asian” (or whichever ancestry) but have grown up as one of the cultural majority and identify as such.

## Data Availability Statement

The raw data supporting the conclusions of this article will be made available by the authors, without undue reservation.

## Ethics Statement

The studies involving human participants were reviewed and approved by Human Research Ethics Committee, University of New England, Australia. The patients/participants provided their written informed consent to participate in this study. Written informed consent was obtained from the individual(s) for the publication of any potentially identifiable images or data included in this article.

## Author Contributions

The three studies reported in this paper were conducted as part of YA's doctoral studies under the supervision of SW. The studies were analyzed and written up by YA for her doctoral thesis. The current submission is a revision of a chapter presented in YA's (unpublished) thesis. The revision, which includes some new analyses, was conducted mostly by SW, but in collaboration with YA. All authors contributed to the article and approved the submitted version.

## Conflict of Interest

The authors declare that the research was conducted in the absence of any commercial or financial relationships that could be construed as a potential conflict of interest.
